# From early sexual debut to later overweight and obesity: a multilevel analysis of Ghanaian women

**DOI:** 10.1186/s41182-025-00753-1

**Published:** 2025-07-01

**Authors:** Joshua Okyere, Castro Ayebeng, Kwamena Sekyi Dickson, Precious Adade Duodu

**Affiliations:** 1 School of Human and Health Sciences, University of Huddersfield, Queensgate, Huddersfield, England, UK; 2 Department of Population and Health, University of Cape Coast, Cape Coast, Ghana; 3 School of Demography, Australian National University, Canberra, Australia; 4 Department of Nursing, School of Human and Health Sciences, University of Huddersfield, Queensgate, Huddersfield, England, UK

**Keywords:** Obesity, Public health, Reproductive health, Sexual debut

## Abstract

**Background:**

Beyond the socio-demographic and lifestyle factors, it is imperative to understand how early-life factors contribute to the development of overweight and obesity. One of such early-life factors is early sexual debut defined as first sexual intercourse before age 16. The study examines the association between early sexual debut and women’s risk of overweight/obesity in Ghana using a nationally representative data.

**Methods:**

Data from the 2022 Ghana Demographic and Health survey was used. We analyzed the data of 6,478 women aged 16–49 years. The outcome variable was obesity status which was computed using the body mass index. The main explanatory variable was early sexual debut. We utilized multilevel logistic regression models to examine the association between early sexual debut and women’s risk of overweight/obesity, controlling for both individual (age, education, internet use, parity, wealth index, anemia status) and community level (residence and ecological zone) confounders.

**Results:**

An estimated 3,183 (48.8% [95% CI 46.9–50.6]) were overweight/obese while 11.7% experienced early sexual debut. After adjusting for individual and community level factors, we found that women with an early sexual debut had a 25% higher likelihood of being overweight or obese compared to those without early sexual debut [AOR = 1.25; 95% CI 1.04–1.51]. The significant confounders associated with higher odds of obesity were increasing age, higher educational level, higher wealth index, parity, and frequent use of the internet. Rural residence and residence in the savannah ecological zone were associated with lower odds.

**Conclusion:**

This study suggests that early sexual debut, a key life course event, may be linked to long-term health consequences including higher risk of overweight/obesity. The results underscore the importance of addressing early-life factors in the prevention of obesity. Public health interventions aimed at delaying sexual initiation could be vital in mitigating the growing burden of obesity in Ghana.

## Background

Globally, the world seems to be moving towards a ‘new pandemic’; the overweight/obesity pandemic. The World Health Organization (WHO) estimates that 2.5 billion adults (18 years and older) were overweight in 2022, of which 890 million were obese [1]. Sex-wise, the global prevalence of overweight is slightly higher among females (44%) than in males (43%) [1]. While the contribution of sub-Saharan Africa (SSA) to the overweight/obesity pandemic is low, its distribution varies across individual countries within the region. For instance, in Kenya, 31.1% of adults are overweight/obese [2]. Nigeria and Ethiopia report a pooled prevalence of 26% and 20% of adults being overweight/obese, respectively [3, 4]. The situation in Ghana is similar to the dynamics in other SSA countries. In a study by Tuoyire [5] the prevalence of overweight/obesity among women tripled between 1993 (6.8%) and 2014 (19.5%), and is projected to increase by 35% by 2040. This makes overweight/obesity a growing public health concern in Ghana.

The extant literature documents the economic and health burden of overweight/obesity. For example, Sweis [6] reports that the economic burden of obesity in the United States to 1603.78 billion dollars when using the disability-adjusted life years (DALYs) approach. Obesity and overweight have been linked to increased risks for non-communicable diseases such as diabetes, stroke, heart disease, hypertension, certain cancers and reduced quality of life [7–9]. Not only does overweight/obesity affect physical health; there is evidence suggesting that it increases the risk of depression by 18 percent [10]. Prior studies [2, 5, 11, 12] have identified some socio-demographic (i.e., being a female, increasing age, urban residency, higher educational attainment, higher wealth index, etc.) and lifestyle factors (e.g., high salt consumption, long screen time, skipping breakfast, etc.) that exacerbate the risk of overweight/obesity. While these risk factors are well-documented, growing attention is being directed toward early-life exposures that may shape long-term health trajectories, including the risk of overweight and obesity. One of such early-life indicators is sexual debut.

Early sexual debut is defined as having “sexual intercourse before 15 years of age” [13]. However, in Ghana, the legal age for sexual initiation is 16 years [14]. While previous studies have explored the link between early sexual initiation and various reproductive (i.e., sexually transmitted infections, teenage pregnancy, unsafe abortions, and later life risky behaviors) [15, 16] and mental health outcomes (e.g., weak sense of coherence, low self-esteem and poor mental health) [17], its relationship with overweight/obesity remains underexplored [18, 19]. There is currently limited evidence on the association between early sexual debut and overweight/obesity, with most existing studies conducted in high-income countries and none from sub-Saharan Africa (SSA), where sociocultural and nutritional contexts differ significantly [18, 19]. One of the few studies conducted in the United States suggests that early sexual debut is associated with 2.5 times higher risk of overweight/obesity [18]. Another study [19] conducted in Seattle found early sexual debut to be significantly associated with poor physical health outcomes including obesity. 

These findings [18, 19], however, emerge from high-income countries with vastly different sociocultural, nutritional, and healthcare systems compared to Ghana, raising important questions about their applicability to sub-Saharan African contexts. As such, the question remains: what is the association between early sexual debut and women’s risk of overweight/obesity in Ghana? Understanding this association within Ghana is particularly relevant for several reasons. First, both early sexual debut and overweight/obesity are emerging public health concerns in the country [5, 14]; second, identifying early-life behavioral predictors of obesity may help inform more holistic interventions that integrate sexual and metabolic health promotion; and third, addressing this link could enhance efforts to meet broader goals related to women’s health and non-communicable disease prevention in low-resource settings. As such, this study examines the association between early sexual debut and women’s risk of overweight/obesity in Ghana using nationally representative data. Specifically, the study estimates the prevalence of early sexual debut and overweight/obesity, as well as examine the association between early sexual debut and overweight/obesity among women in Ghana.

## Methods

### Data source and design

This analysis used data from the 2022 Ghana Demographic and Health Survey (GDHS), specifically utilizing the individual recode (IR) file. The GDHS is part of a broader initiative spanning 85 low- and middle-income countries (LMICs). The primary aim of the 2022 GDHS was to provide up-to-date estimates of key demographic and health indicators. A two-stage sampling approach was employed to accomplish this, yielding a nationally representative sample of 18,450 households across 618 clusters [20, 21]. This method ensured comprehensive coverage at both national and regional levels, representing both urban and rural areas within each of Ghana's 16 regions [20]. 

In the first stage, 618 clusters were selected using a probability proportional to size method, taking into account the urban–rural distribution within each region [20]. Next, a systematic random sampling technique was applied to select clusters in both urban and rural contexts. In the second stage, following the selection of clusters, a thorough household listing and map updating process was conducted, creating a detailed list of households within each cluster. From these clusters, 30 households were randomly selected for interviews. For further details on the GDHS methodology, refer to: https://www.dhsprogram.com/pubs/pdf/FR387/FR387.pdf.

### Measures

#### Outcome

The outcome variable was obesity status which was computed using the body mass index. We computed the BMI for women by dividing their weight (kg) with the square of their height in meters. A BMI of < 24.99 was coded ‘not overweight/obese’ while 25 and above was coded as ‘overweight/obese’ [1, 21]. This categorisation was done to ensure enough cases for analyses and to obtain more robust regression estimates.

#### Exposure

Early sexual debut was the exposure variable. This was computed using the question that asked about the age of the participant when they had their first sexual intercourse. We recoded the raw age into two categories (< 15 years and ≥ 15 years). Sexual initiation before 15 years was classified as having had early sexual debut “yes”.

### Covariates

We controlled for both individual (age, education, internet use, media exposure, parity, wealth index, alcohol use, anemia status) and community level (residence and ecological zone) covariates. These variables were selected based on evidence from existing literature [2, 5, 11, 12] that have found them to be significantly associated with both overweight/obesity and early sexual debut. Media exposure was constructed as an index variable combining respondents who reported reading newspapers or magazines, listening to the radio, and watching television at least once per week. Ecological zones were created based on the region of residence. Western, central, greater Accra and Volta regions were categorised as the coastal zone. The eastern, Ashanti, western north, Ahafo, Bono, Bono east, and Oti regions were classified as the forest zone while the savannah, north east, upper east, upper west and northern regions constituted the savannah zone. Frequency of internet use was based on the question, 'What is the frequency of using the internet in the last month?' with four response options: 'not at all,' 'less than once a week,' 'at least once a week,' and 'almost every day.' For analytical purposes, these were recoded into three categories: 'never,' 'infrequent' (combining 'less than once a week' and 'at least once a week'), and 'often' (representing 'almost every day'). The GDHS by default categorises the anaemia status of women based on their haemoglobin levels. It classifies anaemia into four categories: severe (1), moderate (2), mild (3), and not anaemic (4). For analysis, we recoded this variable into a three-category variable, namely: ‘not anemic’ (coded as 0), ‘mild’ (coded as 1), and ‘moderate/severe’ (coded as 2). This recoding grouped respondents with moderate and severe anaemia due to their relatively low frequencies and comparable clinical relevance [20] (see Table 1).

**Table 1:** Selection of covariates

Variable	Question	Evidence in supporting selection
Age	How old were you at your last birthday?	[2, 5]
Education	What is the highest level of school you attended: pre-primary, primary, middle, JSS/JHS, secondary, SSS/SHS, or higher?	[2, 5]
Internet use	What is the frequency of using the internet in the last month?	[12]
Media exposure	What is your frequency of reading newspaper or magazine; frequency of listening to radio; frequency of watching television?	[5]
Parity	What is the total children ever born?	[5]
Wealth index	Computed based on household amenities (electricity, refrigerator, bicycle, motorcycle, car), main floor material, main wall material, main roof material, type of toilet facility, source of drinking water	[2, 5, 23]
Alcohol use	How many days have you drank alcoholic drinks in the past month?	[1]
Residence	Type of place of residence	[2–4]
Ecological zone	What region were you born in?	[22]
Anaemic status	Anaemia level	[24]

### Statistical analysis

**Fig. 1:**
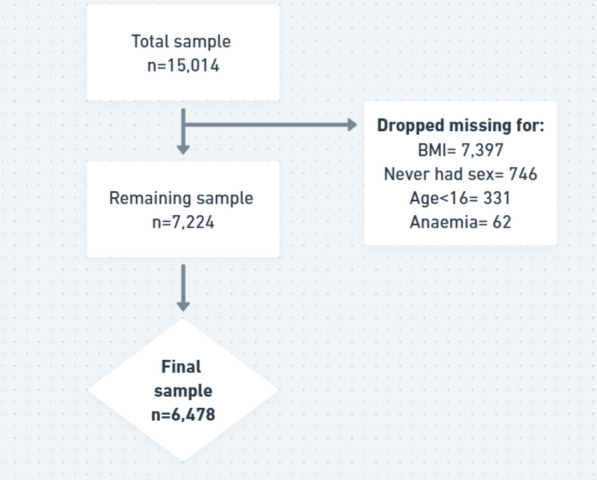
Sample process flowchart

Originally, the 2022 GDHS contains the observations of 15,014 women aged 15–49 years. However, after dropping observations with missing values and those aged 15 years, the sample remained 6,478 women (see Fig. 1). Descriptive analyses were performed to estimate the prevalence of early sexual debut and overweight/obesity among in the sample. We then performed cross-tabulations to assess the distribution of overweight/obesity across the exposure and confounding variables. The results of this cross-tabulation was presented showing the frequencies, percentages, 95% confidence intervals (CI) and corresponding chi-square p-values. The estimates were weighted, and all statistical analyses carried out in STATA version 18 (StataCorp, College Station, TX, USA).

It must be noted that the GDHS is a hierarchical dataset. Hence, a multilevel logistic model was employed to account for the hierarchical structure of the GDHS data, where women (lower level) are nested within clusters (i.e., primary sampling unit) [23]. Women residing in the same community tend to share similar characteristics compared to those in different communities. This violates the assumptions of independence of observations and constant variance across clusters required for the classical binary logistic model.

The null model included only a random intercept to assess baseline variability between clusters and to justify the use of a multilevel approach. Model 1 added early sexual debut to evaluate its unadjusted association with overweight/obesity. Model 2 incorporated individual-level covariates (e.g., age, education, parity) to control for personal characteristics, while Model 3 adjusted for community-level factors (e.g., residence, ecological zone). The final model (Model 4) combined both individual- and community-level covariates to provide a comprehensive assessment.

Covariates were selected based on their theoretical relevance and evidence from prior literature, and their inclusion was evaluated by examining changes in AIC and the statistical significance of predictors. The model with the least AIC values was selected as the model with the best fit. To achieve parsimony, non-significant covariates (p > 0.05) were considered for removal, though their exclusion did not significantly improve model fit.

For the random effects, interclass correlation coefficient (ICC) measure was used to quantify the proportion of total variance attributed to observations (women) within a cluster. This was expressed as:

$$ICC\, = {\raise0.7ex\hbox{${\left( {\sigma^{2} \_u} \right)}$} \!\mathord{\left/ {\vphantom {{\left( {\sigma^{2} \_u} \right)} {\left( {\sigma^{2} \_u\, + \,\sigma^{2} \_\varepsilon } \right)}}}\right.\kern-0pt} \!\lower0.7ex\hbox{${\left( {\sigma^{2} \_u\, + \,\sigma^{2} \_\varepsilon } \right)}$}}\,$$

where:

σ^2^_u = Between-cluster variance.

σ^2^_ε = Between-women variance (follows a standard logistic distribution with variance π^2^/3 and mean 0).

## Results

### Characteristics of study participants

The analysis was based on a weighted sample of 6,478 women aged 16–49 years. Among them, 11.7% reported early sexual debut (before age 15), while the majority (88.3%) did not (Table 2). The age distribution was fairly even, with the largest proportions found among women aged 25–29 (18.2%). Most women had attained at least secondary education (67.4%), while 18.2% had no formal education. In terms of internet use, 38.2% reported frequent use, while 58.0% had never used the internet. Exposure to mass media was high, with 72.8% reporting some exposure. Regarding anaemia status, 22.9% had mild anaemia, and 18.3% had moderate or severe anaemia. In terms of reproductive history, nearly half of the women (45.0%) had three or more children. The wealth distribution was relatively balanced, with 23.6% in the richer quintile and 21.5% in the richest. Urban residents accounted for 56.2% of the sample, while 43.8% resided in rural areas. Regionally, 43.5% lived in the forest zone, 37.8% in the coastal zone, and 18.7% in the savannah zone. Only 15.1% of the participants consumed alcoholic drinks in the past month preceding the survey.

**Table 2:** Prevalence of overweight/obesity among women in Ghana, 2022

Variables	Weighted sample n (%)	Proportion overweight/obese n (% 95%CI)	p-values
Overall	6478 (100.0)	3177 (49.1 [47.2–50.9])	
Early sexual debut			0.653
No	5719 (88.3)	2797 (48.9 [47.0–50.8])	
Yes	759 (11.7)	380 (50.1 [45.1–55.1])	
Age			** < 0.001**
16–19 years	453 (7.0)	75 (16.6 [12.6–21.7])	
20–24 years	1167 (18.0)	360 (30.9 [27.3–34.7])	
25–29 years	1178 (18.2)	554 (47.0 [43.3–50.7])	
30–34 years	1130 (17.4)	641 (56.7 [53.0–60.3])	
35–39 years	1044 (16.1)	640 (61.3 [57.4–65.1])	
40–44 years	852 (13.2)	507 (59.4 [55.2–63.6])	
45–49 years	654 (10.1)	400 (61.2 [56.2–66.0])	
Education			** < 0.001**
No formal education	1177 (18.2)	366 (31.1 [27.9–34.4])	
Primary	932 (14.4)	473 (50.8 [46.3–55.2])	
Secondary/higher	4369 (67.4)	2338 (53.5 [51.3–55.7])	
Frequency of internet use			** < 0.001**
Never	3760 (58.0)	1571 (41.8 [39.7–43.9])	
Infrequent	241 (3.7)	118 (48.9 [40.2–57.7])	
Often	2477 (38.2)	1488 (60.1 [57.1–63.0])	
Media exposure			** < 0.001**
No	1765 (27.2)	637 (36.1 [33.3–39.0])	
Yes	4713 (72.8)	2540 (53.9 [51.8–56.0])	
Anaemia status			** < 0.001**
Not anaemic	3811 (58.8)	2039 (53.5 [51.3–55.7])	
Mild	1483 (22.9)	694 (46.8 [43.4–50.2])	
Moderate/severe	1184 (18.3)	444 (37.5 [33.6–41.5])	
Consumes alcohol			**0.007**
No	5501 (84.9)	2643 (48.0 [46.1–50.0])	
Yes	977 (15.1)	534 (54.7 [50.2–59.1])	
Parity			** < 0.001**
Zero	1298 (20.0)	473 (36.4 [32.9–40.1])	
One	1256 (19.4)	547 (43.5 [39.7–47.5])	
Two	1006 (15.5)	552 (54.8 [50.7–58.9])	
Three or more	2917 (45.0)	1606 (55.0 [52.4–57.6])	
Wealth index			** < 0.001**
Poorest	1068 (16.5)	199 (18.6 [15.9–21.6])	
Poorer	1183 (18.3)	414 (35.0 [31.6–38.5])	
Middle	1309 (20.2)	644 (49.2 [45.9–52.6])	
Richer	1528 (23.6)	928 (60.7 [57.3–64.0])	
Richest	1390 (21.5)	993 (71.4 [67.5–75.0])	
Residence			** < 0.001**
Urban	3642 (56.2)	2179 (59.8 [57.3–62.3])	
Rural	2836 (43.8)	998 (35.2 [32.7–37.7])	
Ecological zones			** < 0.001**
Coastal	2451 (37.8)	1416 (57.9 [54.7–61.1])	
Forest	2817 (43.5)	1445 (51.3 [48.5–54.1])	
Savannah	1210 (18.7)	313 (25.9 [23.5–28.4])	

NB: p-values are computed from chi-square test

Bold indicates statistically significant

### Prevalence of overweight/obesity among women in Ghana, 2022

Of the 6,478 participants, 3,183 (48.8% [95% CI 46.9–50.6]) were overweight/obese (Table 2). The prevalence of being overweight/obese was significantly higher among women who had an early sexual debut (50.1%). The prevalence of overweight/obesity increased significantly with age, ranging from 16.6% among women aged 16–19 years to 61.2% among those aged 45–49 years (p < 0.001). Higher prevalence was observed among urban residents (59.8%), women with secondary or higher education (53.5%), those who often used the internet (60.1%), and those exposed to media (53.9%). Additionally, a higher prevalence of overweight/obesity was found among women who were not anemic (53.5%), parous individuals (55.0% for those with three or more children), those in the richest wealth category (71.4%), and those who consumed alcohol (54.7%). In terms of ecological zones, women in the coastal zone had the highest prevalence (57.9%), followed by those in the forest zone (51.3%) and the savannah zone (25.9%).

### Prevalence of early sexual debut among women in Ghana

**Fig. 2:**
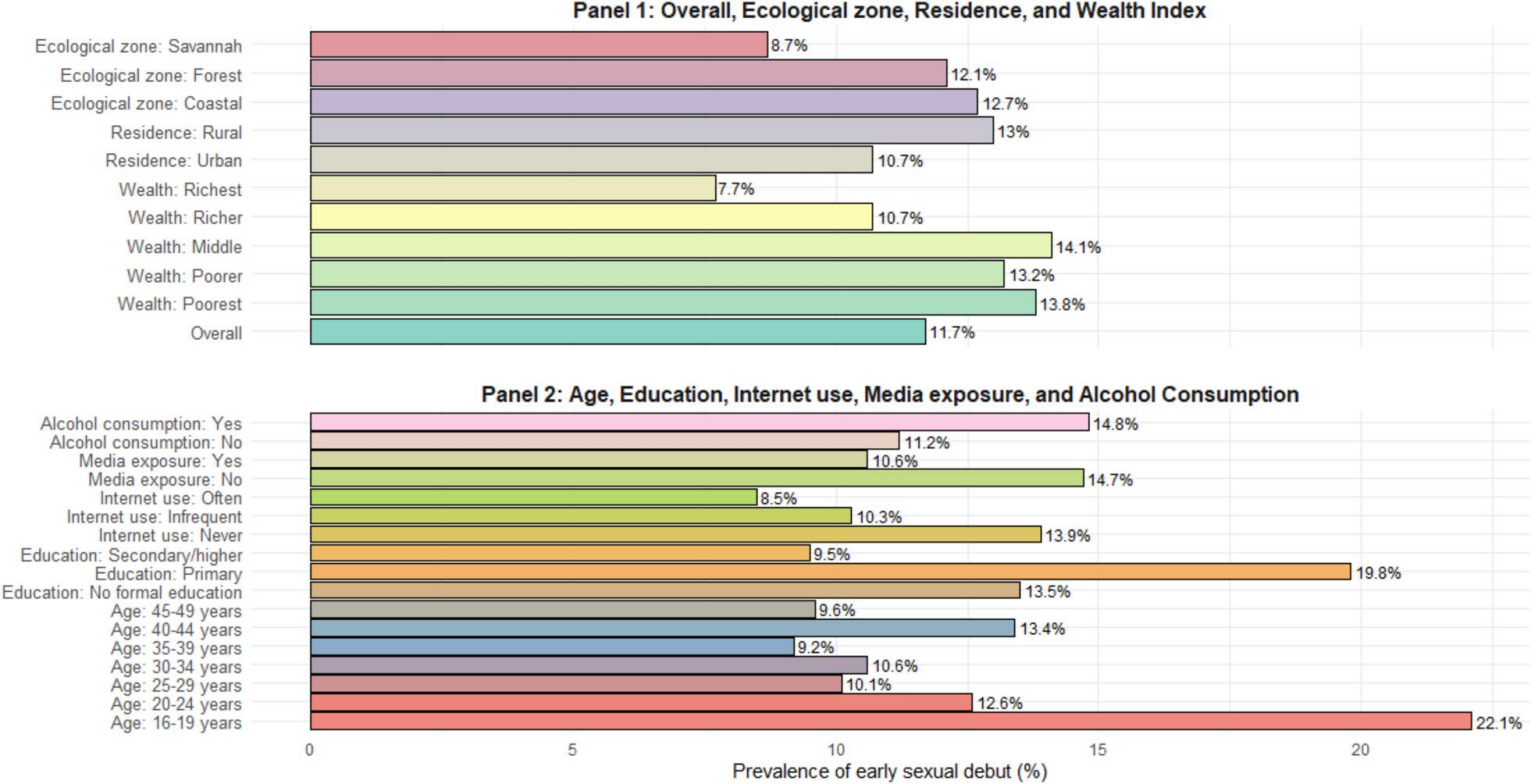
Distribution of the prevalence of early sexual debut among women in Ghana

Overall, 11.7% of women experienced early sexual debut. Early sexual debut was most prevalent among individuals with primary education (19.8%), those who never used the internet (13.9%) and those with no media exposure (14.7%). Additionally, the early sexual debut was prevalent among those who consume alcohol (14.8%), those in the poorest (13.8%) and middle (14.1%) wealth index, as well as among rural residents (13.0%). We found that women aged 16–19 years (22.1%), and those residing in the coastal zone (12.7%) had more cases of early sexual debut (see Fig. 2).

### Association between early sexual debut and overweight/obesity in Ghana, 2022

#### Fixed effects

In Model I, there was no statistically significant association between early sexual debut and overweight/obesity. However, when we adjusted for only individual factors, women who reported early sexual debut had significantly higher odds of being overweight/obese (OR = 1.32, 95% CI 1.10–1.58, *p* < 0.01) compared to those who initiated sex at or after age 15. This association was attenuated and became non-significant in Model III (which adjusted for only contextual factors. However, in Model IV—our fully adjusted model—early sexual debut was statistically associated with overweight/obesity (AOR = 1.25, 95% CI 1.04–1.51, *p* < 0.05), suggesting a persistent relationship after accounting for both individual and contextual factors.

Significant covariates in the full Model included increasing age, with women aged 45–49 years having approximately six times higher odds of being overweight or obese [AOR = 6.32; 95% CI 4.39–9.09]. Higher odds of overweight/obesity were also observed among women with secondary or higher education [AOR = 1.65; 95% CI 1.38–1.96], frequent internet users [AOR = 1.57; 95% CI 1.35–1.84], multiparous women [AOR = 2.02; 95% CI 1.61–2.54], and those in the richest wealth index category [AOR = 5.65; 95% CI 4.32–7.39]. Conversely, rural residents [AOR = 0.81; 95% CI 0.70–0.93] and those living in the Savannah ecological zone [AOR = 0.65; 95% CI 0.54–0.79] had lower odds of being overweight or obese (Table 3).

**Table 3:** Association between early sexual debut and overweight/obesity

Variables	Null model	Model I	Model II	Model III	Model IV
Fixed effects					
Early sexual debut					
No		Ref	Ref	Ref	Ref
Yes		1.02 [0.86–1.22]	1.32 [1.10–1.58]**	0.98 [0.82–1.16]	1.25 [1.04–1.51]*
Age					
16–19 years			Ref		Ref
20–24 years			1.65 [1.21–2.23]**		1.66 [1.22–2.25]**
25–29 years			3.01 [2.20–4.11]***		3.04 [2.23–4.16]***
30–34 years			4.16 [2.99–5.78]***		4.14 [2.98–5.75]***
35–39 years			5.00 [2.99–5.78]***		4.86 [3.46–6.85]***
40–44 years			5.77 [4.07–8.18]***		5.57 [3.93–7.90]***
45–49 years			6.60 [4.59–9.49]***		6.32 [4.39–9.09]***
Education					
No formal education			Ref		Ref
Primary			1.90 [1.56–2.30]***		1.73 [1.42–2.11]***
Secondary/higher			1.84 [1.55–2.17]***		1.65 [1.38–1.96]***
Frequency of internet use					
Never			Ref		Ref
Infrequent			1.08 [0.79–1.47]		1.09 [0.79–1.49]
Often			1.57 [1.35–1.84]***		1.57 [1.35–1.84]***
Anaemia status					
Not anaemic			Ref		Ref
Mild			0.74 [0.65–0.86]***		0.75 [0.65–0.86]***
Moderate/severe			0.56 [0.48–0.65]***		0.56 [0.48–0.65]***
Parity					
Zero			Ref		Ref
One			1.40 [1.15–1.71]**		1.41 [1.15–1.72]**
Two			1.69 [1.35–2.12]***		1.71 [1.37–2.15]***
Three or more			2.01 [1.60–2.52]***		2.02 [1.61–2.54]***
Wealth index					
Poorest			Ref		Ref
Poorer			2.14 [1.77–2.57]***		1.91 [1.58–2.31]***
Middle			3.43 [2.82–4.17]***		2.76 [2.23–3.42]***
Richer			5.05 [4.10–6.23]***		3.94 [3.11–4.97]***
Richest			7.41 [5.82–9.45]***		5.65 [4.32–7.39]***
Residence					
Urban				Ref	Ref
Rural				0.41 [0.36–0.47]***	0.81 [0.70–0.93]**
Ecological zones					
Coastal				Ref	Ref
Forest				0.73 [0.62–0.87]***	0.96 [0.82–1.13]
Savannah				0.32 [0.27–0.39]***	0.65 [0.54–0.79]***
Random effects results					
Variance (SE) of PSU	0.74 (0.08)	0.74 (0.08)	0.10 (0.04)	0.27 (0.04)	0.08 (0.03)
ICC (%)	18.3	18.4	2.8	7.6	2.5
Model fit statistics					
Log-likelihood	− 4223.3314	− 4223.2976	− 3611.4783	− 4070.5667	− 3594.4896
AIC	8450.663	8452.595	7266.957	8153.133	7238.979

Mean variance inflation factor (VIF) = 5.09

*PSU* Primary Sampling Unit, *ICC* Intraclass correlation, *AIC* Akaike information criterion, *SE* Standard error

* p < 0.05, ** p < 0.01, *** p < 0.001

Bold indicates statistically significant

#### Random effects and model fitness

Model IV demonstrated an improved fit compared to previous models, with a log-likelihood of − 3594.4896 and an Akaike Information Criterion (AIC) of 7238.979. The variance component associated with the random effects was 0.08 (SE: 0.03), yielding an intraclass correlation coefficient (ICC) of 2.5%. This indicates that only 2.5% of the variance in overweight/obesity is attributable to cluster-level differences, suggesting minimal unexplained variation at the cluster level and a well-fitted model.

## Discussion

In this study, we examined the association between early sexual debut and women’s risk of overweight/obesity in Ghana using nationally representative data. Our study shows that 25.6 percent of women aged 16–49 years in Ghana had their first sexual intercourse before age 16. The prevalence is similar to Amoako Johnson’s study [25] which estimated an early sexual debut prevalence of 26.7% among women of reproductive age in Ghana. Comparatively, the prevalence of early sexual debut is lower than Nigeria (30.8%) [26]. The difference between our study and that of Yaya and Bishwajit [26] could be the framing of early sexual debut; they used 19 years as the benchmark while we used 16 years. So, obviously, the prevalence would be marginally higher in their study. Our study revealed that the prevalence of early sexual debut was higher among adolescents (16–19 years), rural residents, those from poor households, and women not exposed to media or internet. This is consistent with extant studies conducted in Ghana [27], Malawi [28], and SSA [29].

Nearly half of the participants (48.8%) were overweight/obese. The observed prevalence is higher when compared to Tuoyire [5] study that estimated a prevalence of 19.5% based on the 2014 GDHS. It is also higher when compared to the prevalence in other Anglophone countries in SSA such as Kenya (31.1%) [2] and Nigeria (26%) [3]. This further corroborates Touyire’s projection that Ghana would experience an increase in overweight/obesity [5]. The observed prevalence is a threat Ghana’s vision to achieve the sustainable development goal (SDG) target 3.4 which envisions to reduce premature deaths from non-communicable diseases by one-third by 2030 [30]. With nearly half of the population affected by overweight or obesity, the demand for healthcare services related to NCDs is likely to escalate, exacerbating the challenges of healthcare delivery in a system that is already stretched by infectious diseases and other health priorities.

Consistent with previous studies conducted in the United States [18, 19], we found that women who experienced early sexual debut were 1.3 times more likely to be overweight/obese. A plausible explanation for this association could be that early sexual debut places women at a higher risk of unintended pregnancies which can affect long-term weight trajectories through postpartum weight retention. This is evidenced in a study that showed that women younger than 25 years are more likely to experience postpartum weight retention [31]. We also argue that the observed association may be used to the effect that early sexual debut has on mental health and subsequent coping strategies. There is evidence suggesting that women who experience early sexual debut have a higher risk of suffering psychological distress [32, 33]. In a bid to manage the psychological distress, they may adopt maladaptive coping mechanisms such as unhealthy eating habits which are known to exacerbate the risk of becoming overweight/obese [34]. Nevertheless, our study sets the stage for future studies to explore the moderating and mediating role of psychological distress, postpartum weight retention, and eating habits on the association between early sexual debut and overweight/obesity.

The significant covariates associated with higher odds of obesity were increasing age, higher educational level, higher wealth index, higher parity, and frequent use of the internet. Similar findings have been reported in Kenya [2], Ghana [5] and Denmark [35]. A plausible explanation could be that, higher education may be associated with more sedentary occupations and increased consumption of high-calorie, convenience foods, which contribute to obesity. Similarly, frequent internet user may engage in long hours of sitting and be exposed to prolonged screen time, which can promote weight gain through reduced physical activity [12].

Our study also showed that residing in rural areas reduced the odds of being overweight/obese—a result that resonates with Asosega et al. [36]. The availability of fast food, take-away services, and junk foods are more prevalent in urban areas where such foods have become a culture and to some extent a measure of wealth [37]. It is, therefore, not surprising that women residing in the savannah ecological zone were less likely to be overweight/obese. This zone has the most rural areas in the country and relies heavily on home-cooked, fiber-rich diets that are protective against unhealthy weight gains. It is also possible that the protective association between rural residence/residing in the Savannah zone and overweight/obesity could reflect existing structural inequalities such as food insecurity. A balanced interpretation should be considered [38].

### Implications for policy and practice

The significant association between early sexual debut and increased risk of overweight/obesity, even after adjusting for individual and contextual factors, underscores the need for integrated sexual and reproductive health education that extends beyond traditional concerns like pregnancy and sexually transmitted infections to include long-term physical health outcomes. Policies promoting comprehensive sexuality education should incorporate messaging about the broader health implications of early sexual initiation. Additionally, given the consistent associations found between overweight/obesity and factors such as age, higher parity, urban residence, and socioeconomic status, public health interventions must be multifaceted. For instance, maternal and women's health programs should include weight monitoring and lifestyle counseling, particularly for multiparous and urban women. Moreover, the role of digital and media access (e.g., frequent internet use) suggests an opportunity to leverage mobile and online platforms for targeted obesity prevention messaging.

### Strengths and limitations

This study contributes significantly to the avalanche of empirical evidence on overweight/obesity in Ghana and SSA. The sampling methodology of the GDHS guarantees that the findings are generalizable at the national and district level. Also, the use of a multilevel analytical approach takes care of the clustering biases in the hierarchies of the data. Nonetheless, we cannot establish causal inferences due to the cross-sectional nature of the DHS. Also, the findings may not be applicable in explanation overweight and obesity in younger children (< 15 years) and older women (50 years and older). Given that women had to recall their age at first sex, there is a likelihood of recall bias. The secondary data used does not contain important confounders such as menopausal status, stress level, hormonal conditions, type of diet taken, level of physical activity, other chronic conditions, etc. As such, future studies would have to consider these factors in modelling the association between early sexual debut and overweight/obesity. The use of BMI as a measure of overweight/obesity is another limitation since it does not distinguish between body fat and muscle mass. As a result, women with high muscle mass may be classified as overweight or obese even though they have low body fat. Future studies should consider testing the association between early sexual debut and overweight/obesity using multiple measurements including waist-to-hip ratio, waist circumference and body roundness. 

## Conclusion

This study suggests that early sexual debut, a key life course event, may be linked to long-term health consequences including a higher risk of overweight/obesity. The results underscore the importance of addressing early-life factors in the prevention of obesity. Public health interventions aimed at delaying sexual initiation, alongside strategies that promote healthy lifestyles and weight management could be vital in mitigating the growing burden of obesity in Ghana. An inter-sectoral approach, involving collaboration between the health, education, and social protection sectors, is essential for developing comprehensive strategies to delay sexual debut and reduce the incidence of obesity. This combined effort would ensure that both preventive and supportive measures are in place to foster healthier life trajectories for young people, ultimately contributing to the reduction of obesity prevalence in the country.

## Data Availability

The datasets generated and/or analysed during the current study are available in the Measure DHS repository: http://dhsprogram.com/data/available-datasets.cfm.

## Abbreviations

AIC: Akaike information criterion
AOR: Adjusted odds ratio
CI: Confidence interval
DALYs: Disability-adjusted life years
SE: Standard error
SSA: Sub-Saharan Africa
VIF: Variance inflation factor
WHO: World Health Organization
